# Seroprevalence of anti-SARS-CoV-2 IgG at the first epidemic peak in French Guiana, July 2020

**DOI:** 10.1371/journal.pntd.0009945

**Published:** 2021-11-12

**Authors:** Claude Flamand, Christelle Alves Sarmento, Antoine Enfissi, Sarah Bailly, Emmanuel Beillard, Mélanie Gaillet, Céline Michaud, Véronique Servas, Nathalie Clement, Anaïs Perilhou, Thierry Carage, Didier Musso, Jean-françois Carod, Stéphanie Eustache, Céline Tourbillon, Elodie Boizon, Samantha James, Félix Djossou, Henrik Salje, Simon Cauchemez, Dominique Rousset

**Affiliations:** 1 Epidemiology unit, Institut Pasteur in French Guiana, Cayenne, French Guiana; 2 Mathematical Modelling of Infectious Diseases Unit, Institut Pasteur, UMR2000, CNRS, Paris, France; 3 Laboratory of Virology, Institut Pasteur in French Guiana, Cayenne, French Guiana; 4 Medical Biology Laboratory, Institut Pasteur in French Guiana, Cayenne, French Guiana; 5 Health Centers Department, Cayenne Hospital Center, Cayenne, French Guiana; 6 Clinical Core of the Center for Translational Sciences, Institut Pasteur, Paris, France; 7 Carage Medical Biology Laboratory, Kourou, French Guiana; 8 Laboratoires Eurofins Labazur Guyane, Remire, French Guiana; 9 Aix Marseille University, IRD, AP-HM, SSA, VITROME, IHU-Méditerranée Infection, Marseille, France; 10 Medical Biology laboratory, Centre Hospitalier de l’Ouest Guyanais, Saint-Laurent du Maroni, French Guiana; 11 Infectious and Tropical Diseases Unit, Cayenne Hospital Center, Cayenne, French Guiana; 12 Department of Genetics, University of Cambridge, Cambridge, United Kingdom; 13 Department of Epidemiology, Johns Hopkins Bloomberg School of Public Health, Baltimore, Maryland, United States of America; University of North Carolina at Chapel Hill School of Medicine, UNITED STATES

## Abstract

**Background:**

While Latin America has been heavily affected by the pandemic, only a few seroprevalence studies have been conducted there during the first epidemic wave in the first half of 2020.

**Methodology/Principal findings:**

A cross-sectional survey was performed between 15 July 2020 and 23 July 2020 among individuals who visited 4 medical laboratories or 5 health centers for routine screening or clinical management, with the exception of symptomatic suggestive cases of covid-19.

Samples were screened for the presence of anti-SARS-CoV-2 IgG directed against domain S1 of the SARS-CoV-2 spike protein using the anti-SARS-CoV-2 enzyme-linked immunosorbent assay (ELISA) from Euroimmun.

**Conclusions/Significance:**

The overall seroprevalence was 15.4% [9.3%-24.4%] among 480 participants, ranging from 4.0% to 25.5% across the different municipalities. The seroprevalence did not differ according to gender (p = 0.19) or age (p = 0.51). Among SARS-CoV-2 positive individuals, we found that 24.6% [11.5%-45.2%] reported symptoms consistent with COVID-19. Our findings revealed high levels of infection across the territory but a low number of resulting deaths, which can be explained by French Guiana’s young population structure.

## Author summary

While Latin America has been heavily affected by the pandemic, only a few seroprevalence studies have been conducted there during the first epidemic wave in the first half of 2020.

A cross-sectional survey was performed between 15 July 2020 and 23 July 2020 among individuals who visited 4 medical laboratories or 5 health centers for routine screening or clinical management, with the exception of symptomatic suggestive cases of covid-19.

Samples were screened for the presence of anti-SARS-CoV-2 IgG using the anti-SARS-CoV-2 enzyme-linked immunosorbent assay (ELISA) from Euroimmun.

The overall seroprevalence was 15.4% [9.3%-24.4%] among 480 participants, ranging from 4.0% to 25.5% across the different municipalities. The seroprevalence did not differ according to gender (p = 0.19) or age (p = 0.51). Among SARS-CoV-2 positive individuals, we found that 24.6% [11.5%-45.2%] reported symptoms consistent with COVID-19. Our findings revealed high levels of infection across the territory but a low number of resulting deaths, which can be explained by French Guiana’s young population structure.

## Introduction

The world’s attention remains focused on the spread of severe acute respiratory syndrome coronavirus 2 (SARS-CoV-2), that causes coronavirus disease 2019 (COVID-19), and the implementation of drastic control measures to limit its expansion. By the end of July 2020, more than 17 million confirmed cases and approximately 650,000 deaths have been reported worldwide [1]. With more than 4,500,000 cases and 190,000 deaths, Latin America has been particularly affected by the crisis [1].

A thorough evaluation of the proportion that has already been infected by SARS-CoV-2 and is likely immunized is important to estimate the level of herd immunity of the population [2] and to inform public health decision making. Data on laboratory molecular -confirmed cases do not capture the full extent of viral circulation because a majority of infected individuals have asymptomatic or mild infections and may therefore not seek care [3,4]. In contrast, population immunity is typically estimated through cross-sectional surveys of representative samples using serological tests. Numerous serological surveys were conducted in affected countries during or at the end of the first COVID-19 epidemic wave [5]. Most of the serological studies already available in July 2020 have been carried out in continental Europe [6–12] and in the United States [13–16]. Although Latin America has been heavily affected by the pandemic, only a few seroprevalence studies have been conducted across the continent, meaning the underlying level of infection remains largely unknown [5,17,18]. French Guiana, a French overseas department with 290,000 inhabitants [19], located in Latin America in the Amazonian forest complex experienced a large SARS-CoV-2 epidemic wave. A territory-wide lockdown was set up from March 17^th^ 2020 concomitantly with the rest of French territories, at a time when five imported cases and one secondary case were being confirmed on the territory [20]. The lockdown resulted in limited viral transmission until it was ended on May 11th 2020. In the middle of June there was a rapid intensification of viral circulation over a large part of the territory with 917 confirmed cases of COVID-19 detected from March 4^th^ 2020 to June 11^th^ 2020 [21]. This was followed by the implementation of strict mitigation measures such as curfews and local lockdowns in the course of June and July. The epidemic peaked at the beginning of July with 4,440 cumulative confirmed cases [22], followed by a gradual slowing down throughout the territory. Between March 4 and September 17, 9,623 cases (3,310 per 100,000 inhabitants) of COVID-19 and 65 hospital deaths (22.3 per 100,000 inhabitants) of COVID-19 were detected in French Guiana [23]. As the disease severity is reduced in younger individuals [24], we can suspect that many infections are likely to have been missed in this territory which has a much younger population (mean age of 25,1 versus 32,1 for Latin America and 42,3 for mainland France) [19]. In order to understand the underlying level of infection, we conducted a cross-sectional study within the general population, estimated the seroprevalence of SARS-CoV-2 and assessed its distribution in age groups and geographical areas.

## Methods

### Ethics statement

The study was recorded on Clinicaltrials.gov (ID: NCT04490850) and approved by the “Comité de Protection des Personnes EST-III” Ethical Research Committee (No.CPP 20.07.04–8827; N°ID-RCB 2020-A01826-33). Personal data processing for this study complied with the requirements of the “reference methodology MR-001” established by the French Data Protection Authority (CNIL) regarding data processing in health research. Fieldworker investigators were trained to explain the project, and, when allowed, collect participants’ signatures in a free and informed consent form and carry out the interviews. For participants under 18 years-old, age-appropriate information was given and the written informed consent of legally responsible person was collected. A specific educational-style comic poster was designed to explain, in an understandable way, the nature and objectives of the survey and inform them about the voluntary nature of the participation of the study.

### Study area

**Fig 1:**
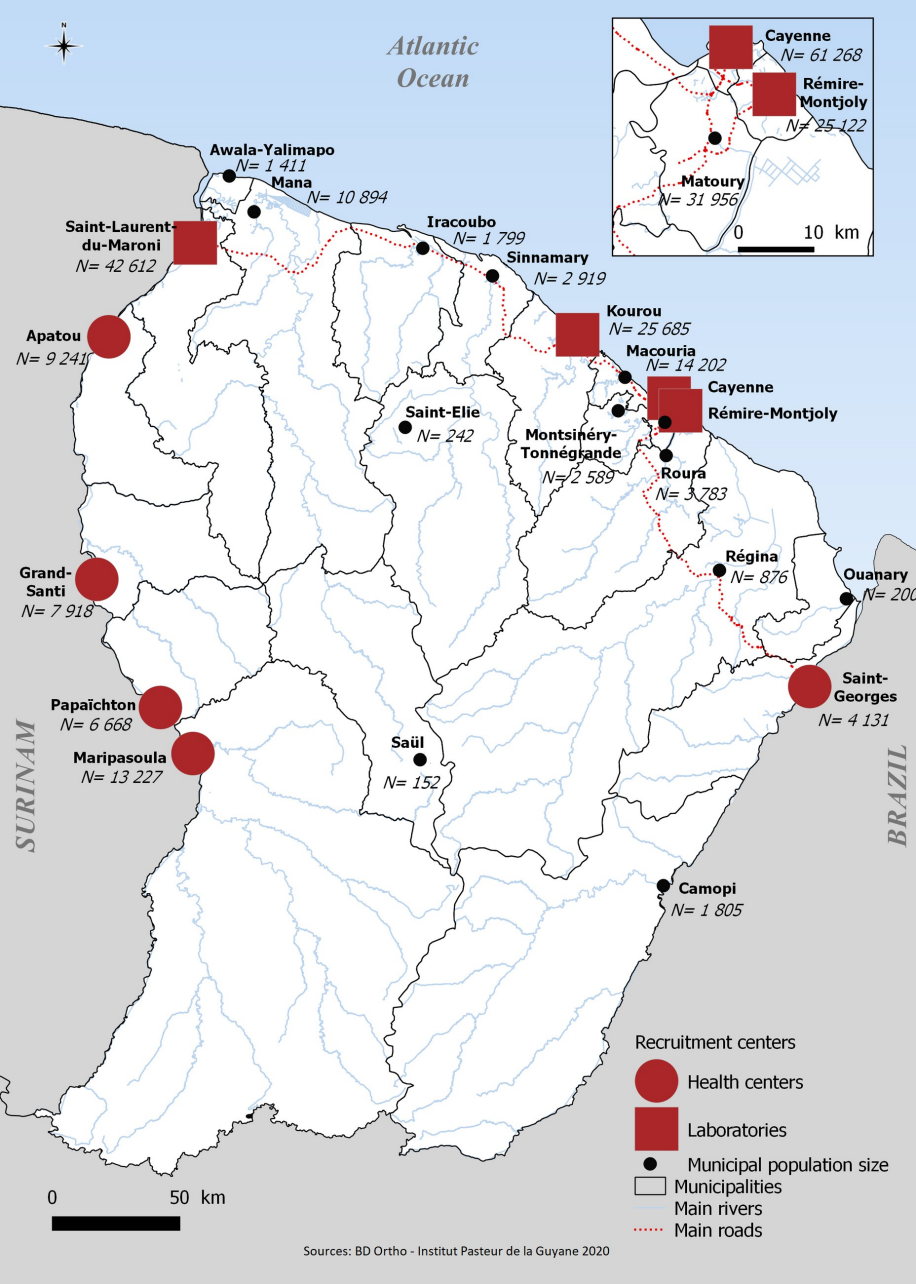
Map of French Guiana with municipal population size and recruitment centers, Geodata source: BD ortho, publicly available at https://www.data.gouv.fr/; QGIS 2.18 software.

French Guiana is composed of two main inhabited geographical regions: a central, urbanized area including a coastal strip along the Atlantic Ocean, where 90% of the population lives, and a more remote area along the Surinamese and Brazilian borders (Fig 1). This territory has the highest crude birth rate in the Americas (25.6 per 1,000 people) and 32% of the population is under the age of 15 [19]. The healthcare system includes 8 medical biology laboratories and 3 hospital centers located in the main municipalities of the coastal area, as well as 17 healthcare centers representing the only health care offer in more isolated areas in the Amazonian rainforest.

### Study design and participants

**Fig 2:**
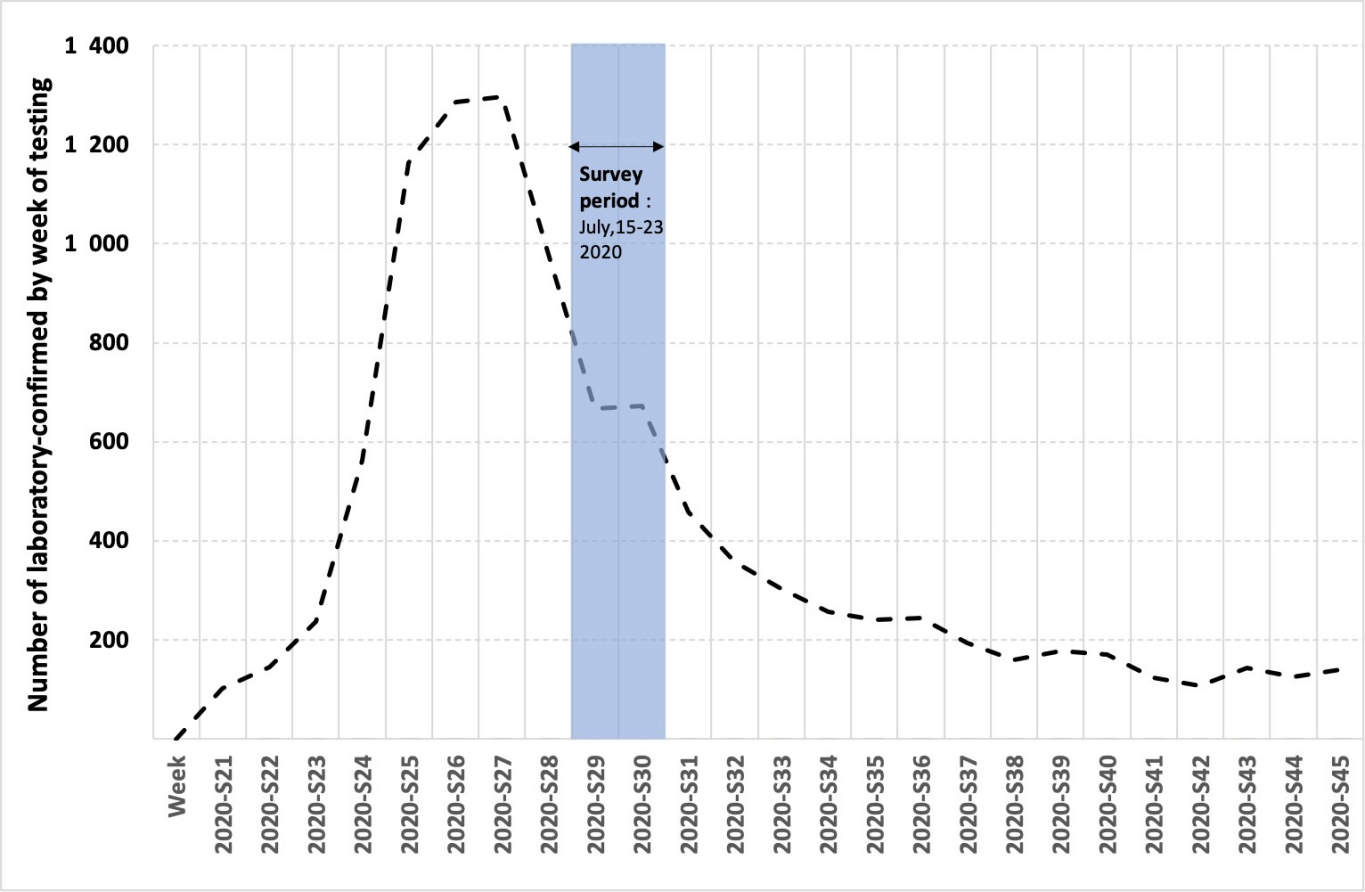
Survey period and temporal evolution of the number of laboratory-confirmed cases, Source SI-DEP, publicly available at https://www.data.gouv.fr/en/datasets.

A cross-sectional survey was performed between 15 July 2020 and 23 July 2020 (Fig 2) in medical biology laboratories located in the coastal urban area of French Guiana and health centers located in isolated areas along the Surinamese and Brazilian borders (Fig 1). All laboratories and health centers were invited to participate in the study. A total of 4 laboratories and 5 health centers had sufficient human and technical resources to implement the study protocol and agreed to participate in the study.

The geographical areas of the different laboratories and health centers involved in the study cover 87% of the population of French Guiana.

Publicity and information about the survey was provided at the reception desk of recruitment centers. All individuals who visited medical laboratories or health centers during the study period for routine screening or clinical management were invited to participate in the study, with the exception of those who came for SARS-CoV-2 viral screening in the context of symptomatic infections potentially suggestive of covid-19.

Demographic data, including age, gender, residential region and occupation of each participant, were collected during a preliminary face-to-face interview through a standardized questionnaire. Participants were asked to report history of symptoms compatible with COVID-19 and to specify if they had consulted a doctor or obtained a biological confirmation of their infection. Thereafter, a venous blood sample of 3.5mL was collected from each participant, in accordance with current biosafety standards.

### Laboratory methods

#### Blood sample collection

Blood samples were collected into 3.5 ml gold BD Vacutainer SST II advance tubes with gel for serum separation (Becton-Dickinson, USA). Immediately after puncture, samples were stored at 4°C-8°C until centrifugation within 12 hours. Sera were then frozen and stored at -20°C until used at the National Reference Center for respiratory viruses in Institut Pasteur in French Guiana.

#### Serologic diagnosis

Collected samples were screened for the presence of anti-SARS-CoV-2 IgG directed against domain S1 of the SARS-CoV-2 spike protein using the anti-SARS-CoV-2 enzyme-linked immunosorbent assay (ELISA) from Euroimmun (Lübeck, Germany). Semiquantitative results were calculated as the ratio of the extinction of samples over the extinction of a calibrator. According to the distributor, the specificity of the test was 99.6%. We internally validated the specificity of the serological assay with serum samples collected from 186 individuals of a cross-sectional serosurvey [25,26] in healthy individuals taken prior to the SARS-CoV-2 outbreak (June to October 2017). Of these, 40.8% were male and the median age was 33.3 years. In this validation subset, the serological test showed a specificity of 97.9% consistent of a recent assessment of this assay [27]. According to the distributor, the sensitivity of the test is 75.0% if the test is performed 10 to 20 days after infection and 93.8% if it is performed more than 20 days after infection.

### Statistical analysis

In order to obtain population representative estimates of overall seropositivity in the territory we weight each sample by the population size within each municipality, age and sex group. We employ the following notation to describe the study design (Table 1):

- *i*: one of the 16 strata (municipalities);
- *M*_*i*_: number of individuals living within the *i*^th^ stratum, *i =* 1, …, 16 (Census data);
- *mi*: number of individuals enrolled from the *i*^th^ stratum, i = 1, …, 16;

We considered that, in each municipality *i*, the probability of selecting a particular subject is equal to (1/*m*_*i*_*/M*_*i*_*)*_*=*_ (*M*_*i*_*/m*_*i*_*)*. This statistical weight indicates the number of people in the population represented by each subject in the sample.

**Table 1:** Weighted SARS-CoV-2 seroprevalence estimated by municipality, July 2020, French Guiana.

Municipalitiy *(i)*	Pop. size	Number of enrolled individuals	Number of SARS-CoV-2-positive individuals	Weighted seroprevalence %[95% CI]
Cayenne	57,614	95	15	25.5% [10.9–48.8]
Matoury	32,427	34	4	11.5% [3.5–32.2]
Saint-Laurent	43,600	20	2	4.3% [1.0–16.8]
Kourou	26,221	95	18	19.8% [8.6–39.1]
Remire-Montjoly	23,976	98	8	4.0% [1.7–9.2]
Macouria	11,719	10	2	20.7% [3.9–62.4]
Mana	10,241	1	0	-
Maripasoula	11,856	13	2	15.2% [2.9–51.7]
Apatou	8,431	22	2	12.1% [2.9–38.5]
Grand-Santi	6,969	40	6	20.5% [8.7–41.1]
Saint-Georges	4,020	29	3	16.1% [4.0–47.3]
Papaïchton	7,266	10	1	7.8% [1.0–50.0]
Sinnamary	2,957	2	0	-
Roura	3,713	8	0	-
Montsinnery-Tonnegrande	2,473	2	0	-
Iracoubo	1,878	1	0	-
Total	259,865	480	63	15.4% [9.3–24.4]

We applied a post-stratification adjustment to each of these weights to arrive at the final statistical weight for each subject. This adjustment helped us to weight the age-sex groups within each municipality to match the distribution in the French Guiana total population. Nine age-groups ([0–15 years[, [15–20[, [20–25[, [20–25[, [25–35[, [35–45[, [45–55[, [55–65[, ≥65 years) were used within males and females groups and for each age-sex subgroups, we applied an adjustment factor *c*_*ijk*,_ to have a final statistical weight

*w*_*ijk*_ *= (M*_*i*_*/m*_*i*_)*c_*ijk*_, where *i* indexes municipalities, j indexes sex groups and k indexes age groups. The outcome of interest was the weighted SARS-CoV-2 seroprevalence estimate.

Analyses were carried out using survey capabilities of Stata version 15 statistical software [28]. French Guiana’s layers were drawn using geodata from the BD Ortho database available from https://www.data.gouv.fr/ under the terms of the CC-BY 2.0 license. Spatial distribution of seroprevalence was mapped using QGIS 2.18 software [29].

## Results

We enrolled 480 participants between July 15 and July 23, 2020, in 16 municipalities (Table 1). The mean age of participants was 38.3 ranging from 0.2 to 87 years old. Comparison of the socio-demographic characteristics of the study sample to the census data demonstrated an over-representation of women (68.1% vs 50% in the general population of French Guiana) and adults over 25 years (79% vs 53% in French Guiana). These differences, mostly related to the structure of the population visiting the laboratories and health centers in French Guiana, were corrected in the analyses of seroprevalence and risk factors by allocating a post-stratification weight to each participant.

Between 15 July 2020 and 23 July 2020, the crude proportion seropositive was 13.1% and the overall weighted seroprevalence of SARS-CoV-2 antibodies in French Guiana was 15.4% [95% Confidence Interval CI, 9.3%-24.4%] (Table 1). This corresponds to 44,660 [95% CI, 26,970–70,760] seropositive individuals out of a population of 290,000.

Since the study was implemented two weeks after the epidemic peak and the sensitivity of the test is limited in the three weeks that follow infection, this likely represents a lower bound for the proportion of individuals infected by the time the epidemic peaked.

The seroprevalence was higher among men than women (19.6% vs 10.4%) but the difference was not significant. There were also no significant differences according to age (p = 0.51) (Table 2).

**Table 2:** Distribution of SARS-CoV-2 seropositivity according to sociodemographic factors, July, French Guiana.

Characteristic	Total tested individuals	Weighted prevalence (%) [95% CI]	Pearson p-value
**Gender**			
Male	153	19.6 [9.7–35.6]	0.19
Female	327	10.4 [6.7–15.9]	
**Age, years**			
[0–9[	16	24.0 [4.7–66.9]	0.51
[10–19[	34	18.7 [4.2–54.4]	
[20–29[	105	9.6 [3.7–22.6]	
[30–39[	125	16.6 [7.6–32.5]	
[40–49[	78	18.4 [9.1–33.5]	
[50–59[	77	9.4 [4.4–19.3]	
[60–69[	34	11.2 [2.5–38.4]	
≥ 70	11	15.2 [4.0–43.6]	
**Geographical area**			
Coastal area	366	15.5 [8.8–25.9]	
Western area	85	14.2 [6.6–27.9]	0.94
Eastern area	29	16.1 [3.9–47.3]	
**Occupational category (over 18)**			
Employed individuals	267	11.8 [7.0–19.1]	0.48
Pensioners	17	4.1 [0.6–25.2]	
Unemployed individuals	156	13.8 [7.7–23.6]	

**Fig 3:**
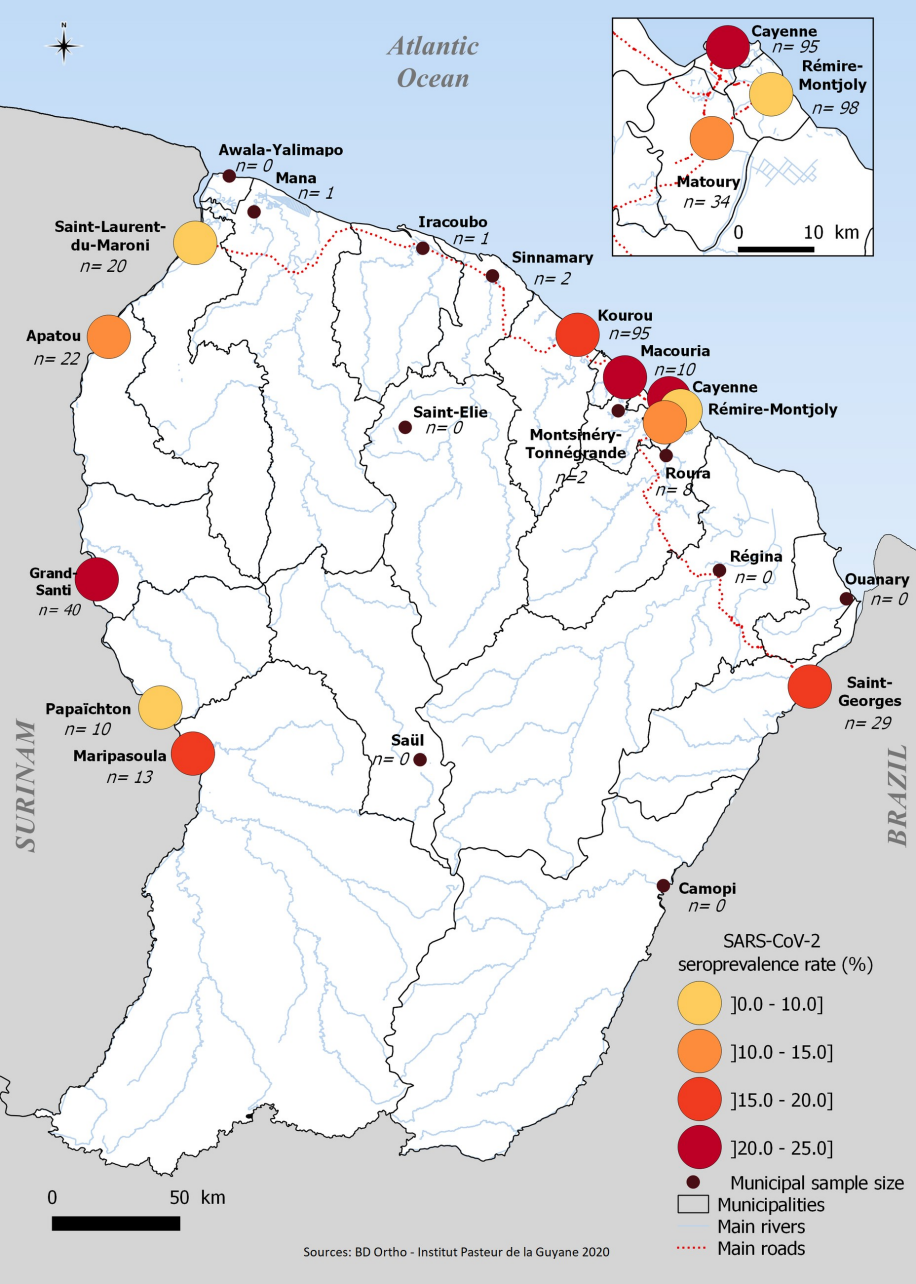
Spatial distribution of SARS-CoV-2 seroprevalence, July, French Guiana; Geodata source: BD ortho, publicly available at https://www.data.gouv.fr/; QGIS 2.18 software.

Serological results in the different geographical areas (Fig 3) showed that SARS-CoV-2 circulated in most of French Guiana (Table 1). Highest infection risks were observed in the main population center in Cayenne municipality (25.5% [10.9% - 48.8%]) in the coastal and urban area. Two other municipalities located in the coastal area were also strongly impacted (Kourou: 19.8% [8.6% - 39.1%] and Macouria: 20.7% [4.0% - 62.4%]). However, even outside the main population centers, some areas appeared to have been heavily affected. For example, among the river areas, Grand-Santi (20.5% [8.7% - 41.1%]), a small municipality located in the western part of the territory and Saint-Georges (16.1% [4.0–47.3]), a municipality located in the eastern part, at the Brazilian border had seroprevalence levels similar to that observed in Cayenne (Table 2).

The proportion of individuals who thought they had COVID-19 was higher among seropositive participants than among seronegative participants (29.9% [14.3%-52.3%] vs 19.9% [14.4%-26.8%]). Among those who reported a presumptive infection, 12.6% [4.9%-28.7%] declared that diagnosis was confirmed by a clinician and 17.1% [6.9%-36.5%] that the diagnostic was confirmed by a SARS-CoV-2 RT-PCR. Nine seronegative participants reported having a RT-PCR confirmation representing 2.1% [0.8%-4.9%] of seronegative participants. Among them, five reported infections less than 3 weeks old and three reported infections older than 3 months.

The presence of compatible COVID-19 symptoms was reported in 24.6% [11.5%-45.2%] of seropositive participants vs 19.5% [14.0%-26.5%] of seronegative participants. Fever (50.7%), anosmia (48.8%), asthenia (48.8%) and cough (34.7%) were the most frequently reported symptoms in SARS-CoV-2 seropositive participants. However, only anosmia was significantly more prevalent in SARS-CoV-2 seropositive *versus* seronegative individuals (p = 0.01) (Table 3).

**Table 3:** Clinical symptoms reported, by SARS-CoV-2 Infection status, July, French Guiana.

Presence of clinical symptoms	Total (N = 102)	Weighted proportion % (CI 95%)	SARS-CoV-2-positive individuals % (CI 95%)	SARS-CoV-2-negative individuals % (CI 95%)	Pearson P value
Fever	56	62.0% [47.0–75.1]	50.7% [23.0–78.0]	64.6% [48.1–78.3]	0.42
Cough	36	28.8% [18.2–42.4]	34.7% [12.1–67.3]	27.4% [16.4–42.2]	0.65
Throat pain	25	17.7% [9.7–30.2]	10.2% [2.7–31.8]	19.5% [10.1–34.3]	0.34
Dyspnea	16	9.6% [4.9–17.8]	7.0% [1.6–25.5]	10.2% [4.8–20.1]	0.63
Arthralgia	36	28.9% [18.2–42.7]	9.8% [2.4–32.4]	33.3% [20.3–49.3]	0.04
Headache	42	26.5% [16.9–38.9]	18.1% [7.1–39.1]	28.4% [17.1–43.1]	0.35
Asthenia	51	45.6% [31.2–60.7]	48.8% [21.8–76.5]	44.8% [28.7–62.1]	0.82
Ageusia	19	26.4% [14.6–42.8]	25.4% [9.7–51.6]	26.6% [13.3–46.2]	0.92
Anosmia	16	15.1% [7.6–27.7]	48.8% [21.8–76.5]	7.3% [2.4–20.4]	0.01
Diarrhea	23	21.4% [11.4–36.7]	31.6% [10.1–65.6]	19.1% [8.9–36.5]	0.42
Vomiting	4	1.0% [0.3–3.1]	2.9% [0.7–11.5]	0.5% [0.1–3.8]	0.13

## Discussion

We report the first serosurvey for the detection of SARS-CoV-2 antibodies in French Guiana. We found that 15.4% [9.3%-24.4%] of the population was seropositive two weeks after the peak of the first epidemic wave. Assuming a two or three-week delay for seroconversion, our estimation reflects the level of infection of the population at the end of June or beginning of July, which roughly corresponds to the epidemic peak. Our results indicate that by that time, at least 44,660 [26,970–70,760] of French Guiana’s 290,000 population had been affected by the virus, more than 10 times the official count of 4,440 confirmed cases reported by public health surveillance system by the first week of July [22]. Various investigators have shown that seroprevalence levels in South American populations during the first epidemic wave were much higher than those reported in European cities and countries [30]. It appeared in countries such as Peru, Colombia, Argentina and Brazil, that the highest values came from low-income populations. The relationship with poverty has already been demonstrated in different studies [30]. In Brazil, one of the bordering countries in French Guiana, seroprevalence estimates varied markedly across the country’s cities and regions, from below 1.0% in most cities in the south and center-west regions to up to 25.0% in the city of Breves in the Amazon (North) region [17]. Nevertheless, overall seroprevalence was estimated at 1.4% (95% CI 1.3–1.6). In contrast, our findings highlighted high but also relatively homogeneous levels of infection in most municipalities, ranging from 10% to 20%.

The case fatality rate of COVID-19 was low during the outbreak as there were 65 COVID-19 related deaths from the beginning of the outbreak up to September 17 [23] across the territory while about 45,000 people have been infected at the beginning of July. This was probably due in part to the young age of the population of French Guiana.

Younger populations are likely to have more social ties than older populations, and therefore physical distancing may be more difficult to implement than in countries with ageing populations. Furthermore, since young people are less susceptible to disease severity, they may be less likely to adopt physical distancing measures when they are infected and potentially contagious in a context of high level of transmission [31].

Seroprevalence was higher in men than in women (19.6% versus 10.4%), which could reflect a different exposure potentially related to a greater number of contacts and exposure situations in men than in women but this difference was not significant in our study. Although several population-based studies have demonstrated differences in seroprevalence rates between male and female subjects, most of them indicate that seroprevalence does not differ significantly between men and women [32].

Our study has several limitations inherent in the study design. Our approach made it possible to obtain rapid estimates of the impact of the epidemic. However, convenience sampling may result in a lack of population representation if part of the general population has lower access to the laboratories and health centers participating in the study. In our study, we observed a significant under-representation of men and children under 15 years of age, related to the structure of the population typically visiting laboratories and health centers, and therefore performed a post-stratification adjustment. However, this may have led to large confidence intervals for some of parameter estimates. In addition, sample size calculation was determined to obtain a sufficient point estimate of territory-wide prevalence estimates but not to study risk factors of infection. A few municipalities with no laboratory or health centers were not represented. However, the municipalities represented by the laboratories and health centers involved in the study are home to 90% of the population, so that our estimates are likely a good reflection of the situation across the territory.

We may underestimate infection levels if precarious populations are at higher risk of infection and have limited access to health facilities. Furthermore, it is also possible that sampled individuals who were routinely monitored for pregnancy or chronic health problems took more precautions and reduced their exposure to the virus.

Although it is difficult to assess the representativeness of the sample, the relatively easy access to health care and diagnostic facilities in Guyana, particularly in the larger population centers represented in our study, may have limited coverage bias.

Additionally, it is possible that infected people did not develop specific SARS-CoV-2 antibodies or that these antibodies were not detected by our assay. Since the study was performed shortly after the peak of the epidemic, a proportion of individuals infected at the peak may not have seroconverted by the time of sample collection. With the exception of anosmia, which is already known as a common and distinctive features of SARS-CoV-2 infection [33,34], symptoms were not significantly more frequently reported by seropositive than seronegative individuals.

In conclusion, we found that at least 15.4% of the population in French Guiana was infected by SARS-CoV-2 by the time the epidemic peaked in July. Our estimates are close to the infection attack rate of SARS-COV-2 of 17.6% [17.2%, 18.0%] estimated in a recent modeling study conducted to characterize the epidemic dynamics and to evaluate the impact of control measures that were implemented to contain the epidemic in French Guiana [35].

This corresponds to an elevated infection burden given the relatively limited mortality, which can be explained by French Guiana’s young population structure.
